# A Questionnaire-based examination of the validity of the tourism climate index for application in South Africa

**DOI:** 10.1007/s00484-025-02965-w

**Published:** 2025-06-23

**Authors:** Carmen K. Kganane, Jennifer M. Fitchett

**Affiliations:** School of Geography, Archaeology and Environmental Studies, University of the Witwatersrand, Johannesburg, South Africa

**Keywords:** Tourism, Climate, Questionnaires, Preferences, Climate variables

## Abstract

Tourism Climate Indices have been developed over the past four decades to quantify and classify the climatic suitability of a given destination. However, their development and testing has primarily been conducted in the Global North. The Tourism Climate Index (TCI) was developed based on subjective judgement of scientists, the Holiday Climate Index (HCI_urban and beach_) on the preferences of tourists in Europe, and the Camping Climate Index (CCI) on camping occupancy in the USA. To ascertain whether a particular index is suitable for application in more distal regions it is crucial to test the local validity of the index. The TCI has previously been applied in 10 tourist locations across South Africa, and the validity tested against TripAdvisor reviews. In this study, the suitability of applicable tourism climate indices are tested against 870 survey questionnaires completed by tourists across the same cities. The questionnaire responses reveal that the importance of each climatic variable differs between cities, and for local versus international tourists. The results also demonstrate that thresholds of unacceptable temperature, likewise, vary by city and tourist’s country of origin. However, given the broad alignment with the weightings of the aforementioned tourism climate indices, rather than adjusting the individual indices based on these results, we argue for a more careful interpretation of the output scores and how they relate to the varied experiences of tourists within a location.

## Introduction

Globally, “sunny South Africa” is the primary framing for the advertising and identity of the country for tourism (Henama et al. 2016). As much of the country is located within the summer rainfall zone, where storms are predominantly convective in nature and short lived, South Africa does indeed experience long sunny days year-round (Lennard 2019). However, sunshine is not the only factor that determines the climatic suitability for tourism. The rainfall, wind speed and thermal comfort of a region contribute to the types of activities on offer, the seasonality optimal for tourism, and the competitive advantage relative to otherwise similar destinations (Conradie 2012). The weather during a vacation further influences a tourists’ enjoyment, wellbeing, and the ability to take part in planned activities (Denstadli et al. 2011).

To quantify the climatic suitability of a destination, a range of indices and rating systems have been developed, using meteorological input data to compute and classify attraction-specific suitability (de Freitas et al. 2004). The first of these was Mieczkowski’s (1985) Tourism Climate Index (TCI), which had been developed to evaluate the climatic suitability for world tourism. This index has been widely applied in regions as diverse as the Mediterranean (Amelung and Viner 2006), the Canary Islands (Alonso-Perez et al. 2021), China (Yu et al. 2021), Turkey (Adiguzel et al. 2022), Georgia and Armenia (Amiranashvili et al. 2014), and southern Africa (Noome and Fitchett 2019; 2021; Fitchett et al. 2017; Mushawemhuka et al. 2021). However, critiques of the index have been raised on the grounds that it developed based on the subjective judgements of experts, without considering the climatic experiences of tourists (de Frietas et al. 2004). This prompted the development of a range of questionnaire-informed tourism climate indices, including the Holiday Climate Index for urban environments (HCI _urban_; Scott et al. 2016) using tourist preference data from six European cities, and the Holiday Climate Index for beach environments (HCI _beach_; Rutty et al. 2020) based on tourist arrivals data in the Caribbean. The Camping Climate Index (CCI; Ma et al. 2020) was developed based on campsite occupancy data. However, these are each sector-specific indices, and thus all four indices remain widely used (Saarinen 2013). Increasingly, these indices are being applied at a global scale, or in regions far from where they were initially developed. Therefore, it is beneficial to explore the validity of the index in the context of tourist experiences of weather, especially in a location distal to its development. This has been conducted in South Africa for the CCI using survey questionnaires (Meyer and Fitchett 2023), and for the TCI using TripAdvisor reviews (Fitchett and Hoogendoorn 2018, 2019).

This study uses a large sample of questionnaire responses to explore the validity of the TCI, and indeed the other locally relevant indices, for application in South Africa, through exploring the relative importance of meteorological variables to tourists across the 10 cities, and the thresholds for what is considered to be unsuitable weather.

## Methods

### Study site

**Fig. 1:**
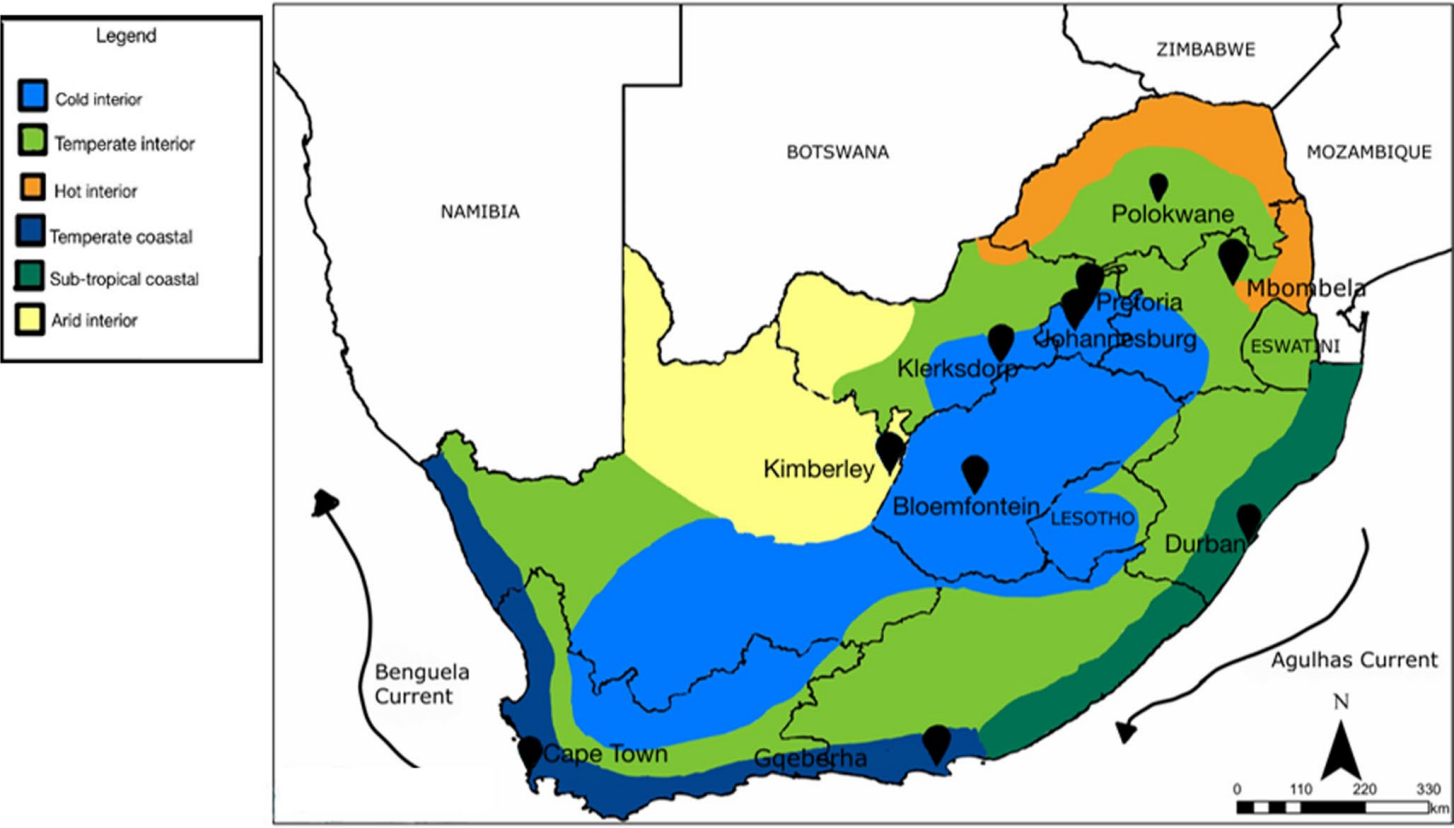
Map of South Africa demonstrating the 10-study locations, with accompanying climate zones and oceanic currents

South Africa is the southern-most country in Africa spanning 22°S-35°S and 17°E-33°E, with an altitude ranging from 0 m.asl along the extensive coastline, to 3,400 m.asl in the Drakensberg Mountains (Lennard 2019). As a result of the latitudinal and altitudinal range, and proximity to the warm Agulhas current to the east and cold Benguela current to the west, the country experiences a variety of climatic conditions (Fig. 1). This vast variation in climate coupled with the beaches, nature reserves and numerous outdoor activities, makes South Africa a suited destination for tourism, (Fitchett and Hoogendoorn 2018). Tourism in South Africa is also a viable source to the growth of the economy with both international and domestic travel (Rogerson and Rogerson 2017).

For this study, 10 locations were selected for the in-person distribution of survey questionnaires, namely Johannesburg, Pretoria, Polokwane, Mbombela (formerly Nelspruit), Durban, Gqeberha (formerly Port Elizabeth), Cape Town, Bloemfontein, Kimberley and Klerksdorp. The primary selection factor was the previous calculation of the TCI (Fitchett et al. 2017), and the initial validation against TripAdvisor data (Fitchett and Hoogendoorn 2018, 2019). These locations span the nine provinces of South Africa, and the different climatic zones (Fig. 1). They offer a wide range of touristic attractions spanning a number of the categories of tourism as defined by UN Tourism (2025), including beach tourism, business tourism, and heritage and cultural tourism (Table 1).

**Table 1:** The 10 study sites in South Africa with their primary tourism attractions, as categorised by the UN tourism (2025) glossary of terms

City	Tourism Attraction (Fitchett et al. 2017)	TCI Scores (Fitchett et al. 2017)
Johannesburg	Business and Adventure Tourism	85.2
Pretoria	Business, Heritage and Cultural Tourism	87.5
Polokwane	Nature-based and Heritage and Cultural Tourism	86.8
Klerksdorp	Nature and Heritage and Cultural Tourism	Not calculated
Durban	Coastal and Historical Tourism	84.2
Gqeberha (formerly Port Elizabeth)	Coastal and Nature-based Tourism	80.2
Cape Town	Business and Adventure Tourism	83.5
Kimberley	Nature-based and Heritage and Cultural Tourism	87.4
Bloemfontein	Historical and Heritage and Cultural Tourism	84
Mbombela (formerly Nelspruit)	Lifestyle, Leisure and Nature-based Tourism	87.1

## Data collection and analysis

This study is the first to use survey questionnaire data to explore the validity of the TCI and HCI for use in South Africa. This study draws on the approach of Fitchett and Meyer (2023) in evaluating the validity of the CCI for South Africa from survey questionnaires, with a considerably larger sample of 870 completed responses. It furthers the work in determining the validity of the TCI through TripAdvisor reviews (Fitchett and Hoogendoorn 2018, 2019). The responses from these questionnaires are used in confirming the validity of all four tourism climate indices that have been applied in South Africa.

Questionnaires were distributed to tourists in each of the 10 cities in person outside of popular tourist attractions, as hardcopy surveys. A convenience sampling approach was used, identifying tourists who were waiting for activities to commence and were thus willing to take part in completing the questionnaire. The survey questionnaire comprised 33 questions which included demographics of the respondents, and their climatic preferences. Tourists were asked to rank the relative importance of each meteorological variable, and to state the threshold conditions under which they considered the climate to be unsuitable for tourism.

The analysis of the questionnaire responses included the quantification of the number of times each variable of the TCI had been included in the dataset. This method had been used before to assess the climate suitability of tourist destinations by analysing the effectiveness of the TCI in accordance with TCI ratings compared to observed tourist activities (Fitchett and Hoogendoorn 2019). This study also used the questionnaire responses as a determinant of climatic variables of greatest importance to tourist. The climatic variables included precipitation, daytime temperature, night-time temperature, windspeed, humidity, cloud cover, and sunshine. Participants ranked each climate variable as either not a consideration’, ‘little importance’, ‘neutral’, ‘important’ or ‘very important’. The level of ranking for each variable was compared to the percentage of people who indicated how important each TCI variable is. Based on this comparison, a revised weighting system for each variable was developed, different from the original weightings of TCI variables. For the final analysis of the data, tourists’ temperature thresholds had been determined for unacceptably hot and unacceptably cold temperatures.

## Results

Of the total 870 questionnaire responses, 282 responses were from international tourists, while 588 were local, South African tourists. However, each study site had a varying distribution of questionnaire responses. For Bloemfontein, only 36 responses obtained, and for Klerksdorp a similarly low 42 questionnaires were completed. A total of 80 questionnaires were completed in Gqeberha, and 87 in Kimberly. For Durban, Pretoria and Johannesburg, 100 responses were obtained for each city. For Polokwane and Mbombela a similar number of responses were obtained, at 101 and 103 respectivelyThe largest number of responses were obtained for Cape Town at 121,

## Ranking of climate variables’ importance for South African and international tourists

**Fig. 2:**
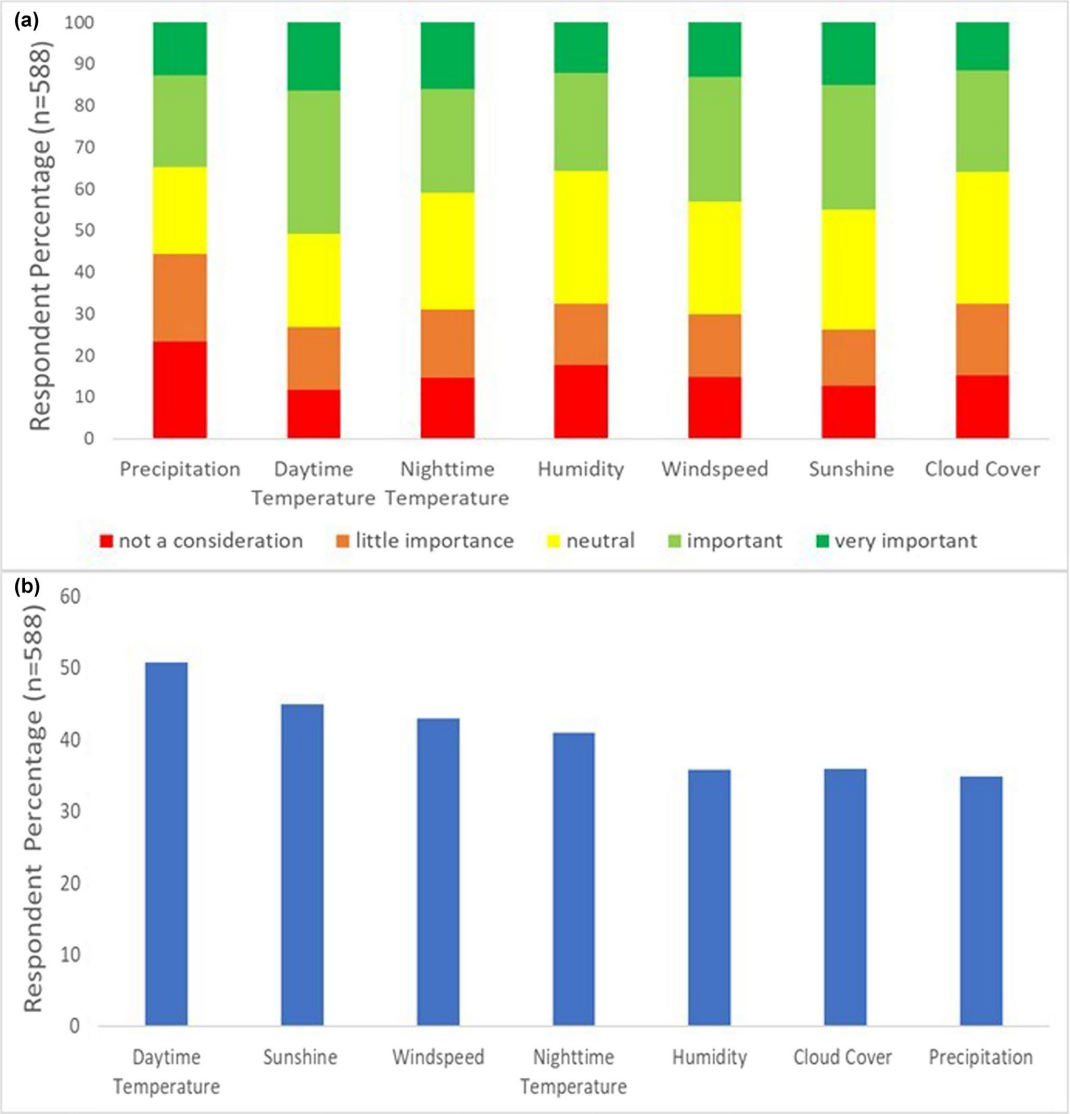
South African participants perceptions of the relative importance of meteorological variables, (a) for all rankings of importance, and (b) the cumulative total of rankings ‘important’ and ‘very important’

The responses from the questionnaires demonstrate a range in the level of importance participants ranked each variable for the 590 respondents who answered this question within the questionnaire (Fig. 2a). Daytime temperature is the highest ranked variable for the level of importance with 50% of the participants classifying it as very important, compared to precipitation, which is the lowest ranked variable for South Africa with only 45% of the participants classifying it as very important (Fig. 2b). For the cumulative ranking for South Africa, precipitation had a higher level of respondents who ranked the variable as ‘not a consideration’ which then suggests that this variable may have not been a significant factor for tourists as more than 20% of participants classified it as such (Fig. 2a). In comparison to the other climate variables, daytime temperature had more respondents ranking the variable as ‘important’, highlighting daytime temperature as a key environmental factor in travel decisions with 30% of the participants classifying the variable as ‘important’ (Fig. 2a).

**Fig. 3:**
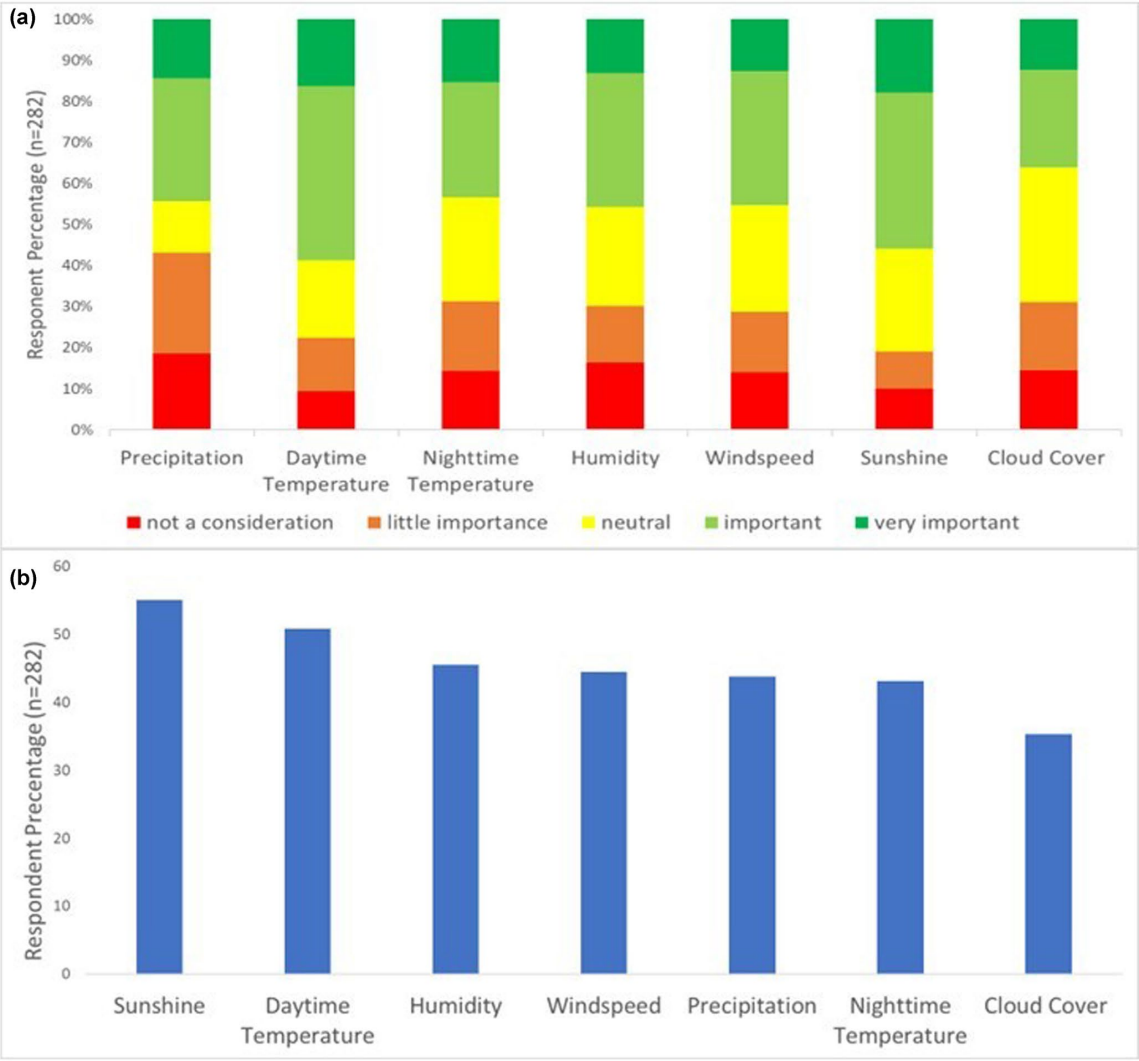
International participants perception of the relative importance of meteorological variables, (a) each ranked in terms of importance, and (b) comparing the total ranking of ‘important’ and ‘very important’

Classifications and rankings had also been explored for international tourists. International tourists consider sunshine to be a variable of greater importance as the respondent percentage for ‘not a consideration’ is lower than the combined respondent percentage for ‘important’ and ‘very important’ (Fig. 3a).

There had also been a higher respondent percentage of international tourists who classified cloud cover as ‘neutral’ compared to all the other variables (Fig. 3a). Daytime temperature has a lower respondent percentage who classified the variable as ‘important’, compared to sunshine (Fig. 3a). However, the level of ranking for ‘important’ and ‘very important’ for sunshine (65%) is higher than that of Daytime temperature (51%), which means that international tourists are more concerned about the number of sunshine hours in South Africa, than the daytime temperature (Fig. 3b). Cloud cover was the least important climate variable tourists were concerned about as the total respondent percentage who classified the variable as “important” and “very important” is at 46% (Fig. 3b).

## Ranking of climate variables’ importance for each tourist location

For each of the 10 different tourist locations, precipitation, daytime temperature, night-time temperature, humidity, windspeed, sunshine and cloud cover were ranked by respondents according to their perceived level of importance. The trend in the ranking levels vary from city to city depending on the geographical location of the city or the type of activity tourists would participate in.

**Fig. 4:**
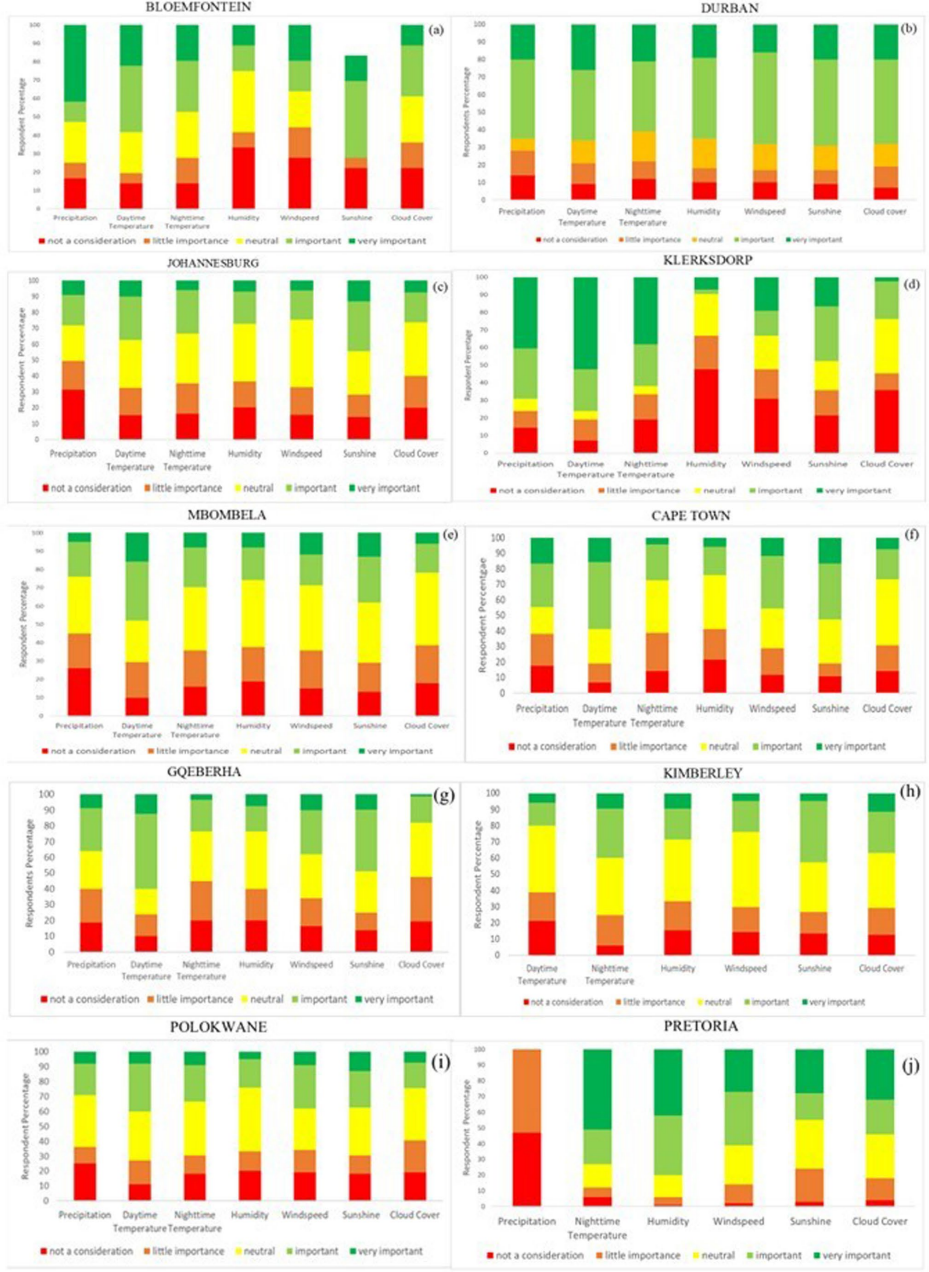
TCI variable classifications according to the respondent percentage that classified each variable as ‘not a consideration’, ‘little important’, ‘neutral’, ‘important or ‘very important’ for (a) Bloemfontein, (b) Durban, (c) Johannesburg, (d) Klerksdorp, (e) Mbombela, (f) Cape Town, (g) Gqeberha, (h) Kimberley, (i) Polokwane and (j) Pretoria

However, there are a few cities that follow a similar trend in terms of the ranking levels, where certain variables from the TCI would have the same respondent percentage classifying it as a certain level of importance. The cities of Johannesburg, Mbombela, Cape Town, Kimberly and Polokwane all have a similar trend in how respondents ranked each variable. While the city of Durban has a high discrepancy in how the variables had been ranked, where there is a large proportion of respondents who perceive each variable to be important (Fig. 4). Dissimilar to the other cities, Klerksdorp has the highest level of ranking deemed ‘not a consideration’ for humidity (Fig. 4). This is also in contrast to Pretoria, where there is no ranking for humidity being ‘not a consideration’ (Fig. 4). Pretoria is also significant with the ranking of precipitation, where the variable is not deemed as important because there had been no respondent percentages recorded for the ‘important’ or very important’ classifications (Fig. 4).

From these results, further examination on the climate variables tourists deemed as important to their stay or activity can be made. All the TCI variables were important to tourists when choosing to visit the city of Durban. Furthermore, in the cities of Cape Town, Mbombela and Gqeberha, daytime temperature of greater importance to tourists than any other climate variable of the TCI (Fig. 4). The city of Pretoria had no record of precipitation being ‘important or very important, which means that tourists did not consider the likelihood of rain to be important during their stay in Pretoria (Fig. 4). 

### Temperature thresholds for South Africa and each tourist City

**Fig. 5:**
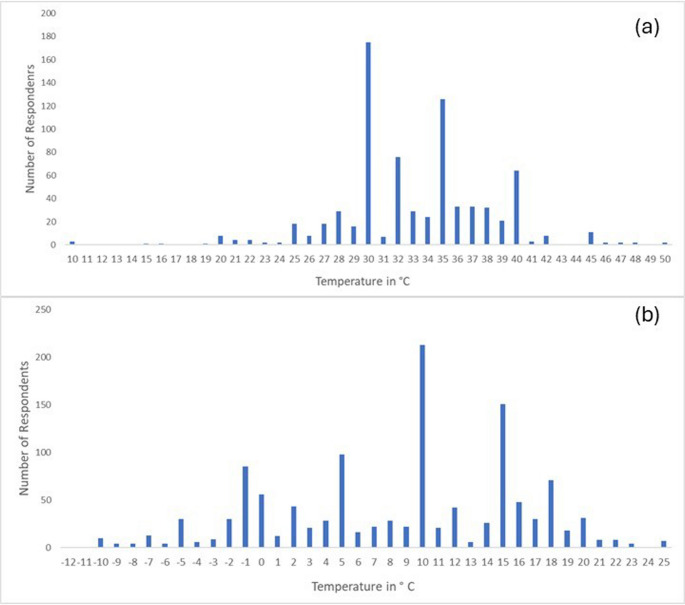
The overall temperature thresholds for both South African and international tourists for hot (a) and cold (b) temperatures

The total number of respondents who indicate certain temperatures as being unacceptably hot or unacceptably cold ranges differs between the group of South African tourists and international tourists. There is also a city-to-city range where participants within each city have different thresholds. Each graph follows a similar trend where a range of temperature thresholds are given, and a peak in the temperature at which most respondents say is their threshold is displayed. When considering demographics, for the thresholds for unacceptable temperatures, international and local respondents have the same modal answer (Fig. 5).

**Fig. 6:**
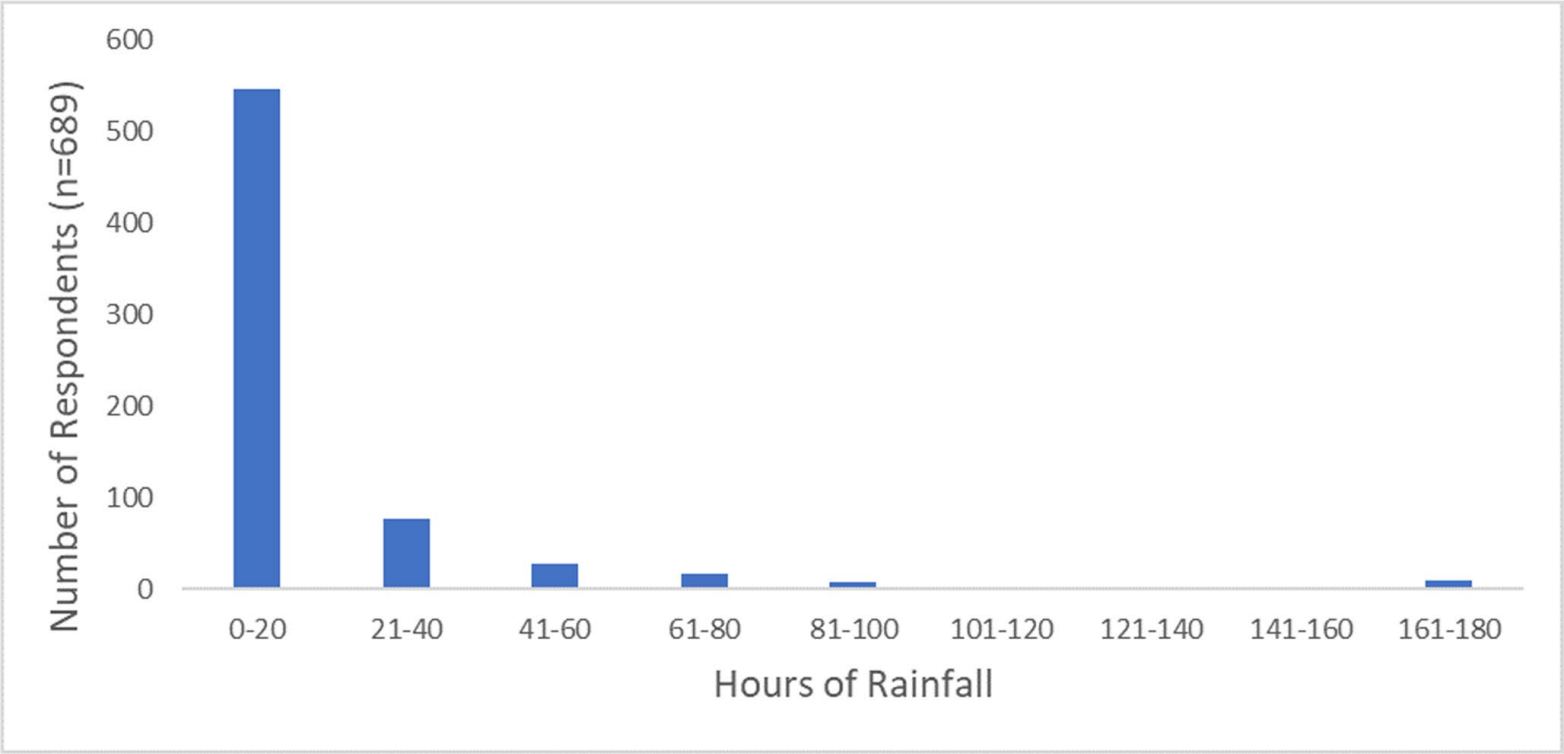
Consecutive hours of rainfall tourists deem as unacceptable

Furthermore, an analysis of the precipitation thresholds the number of consecutive hours of rainfall that tourists consider to be unacceptable to the climatic suitability of a destination for their intended tourism (Fig. 6). The majority of the participants agreed that between 0 and 20 h of consecutive rainfall is their threshold (Fig. 6).

There are also temperature thresholds for each city, where cities may have multiple unacceptably cold temperature thresholds like Kimberley and Durban. Table 3 gives a summary of the thresholds for each of the 10 tourist locations, where it is illustrated that one city will have multiple thresholds for unacceptably cold temperatures (Table 3). It is also illustrated that multiple cities will have the same threshold for both unacceptably cold and hot temperatures (Table 2). Although there had been a lot of variability in the variables of importance between the cities, when it comes to the thresholds, the temperatures were similar.

**Table 2:** Hot and cold temperature thresholds for each of the 10 tourist cities as well as the thresholds for precipitation in hours

City	Cold Temperature Threshold (°C)	Hot Temperature Threshold (°C)	Precipitation Threshold (Hours)
Bloemfontein	0	30	3 h
Durban	−2; 10	30	4 h
Johannesburg	10	30	2 h
Klerksdorp	−1	30	5 h
Mbombela	10	35	3 h
Cape Town	10	30	3 h
Gqeberha	15	30	24 h
Kimberley	−1; 5; 10; 15	30	2 and 4 h
Polokwane	15	30	2 h
Pretoria	10	40	24 h

## Discussion

The key difference between the different tourism climate indices is the relative importance placed on different meteorological variables; through the weightings these are allocated in the equations. The weightings of each variable do differ from each equation, depending on the importance of the variable to the specific location or activity taking place. The HCI_urban_ consists of similar climate variables to the TCI, however sunshine hours is replaced by (A) aesthetic which is quantified instead by the amount cloud cover (Scott et al. 2016). The weighting of day thermal comfort variable in the HCI_urban_ is the same as that of the TCI however, in the TCI the 24-hour thermal comfort accounts for a further 10% (Scott et al. 2016). Similarly, the weighting combination of the wind and precipitation also equals 40% which ensure that a high HCI_urban_ score is not obtained through the dominance of thermal comfort, which is weighted as less important than the physical climatic variables (Scott et al. 2016). The weighting of precipitation decreases by 10%. For the HCI_beach_, thermal comfort decreases by 20% as majority of the beach tourists ranked the variable as 3rd most important, again with nighttime thermal comfort excluded, while cloud cover was ranked as the most important, hence the 40% weighting (Rutty et al. 2020). The reduction in thermal comfort weighting is with the basis that beach tourism is a daytime activity, and the availability of air-conditioning is universal (Rutty et al. 2020). The weightings of climate variables in the CCI for daytime thermal comfort is 10% more than that of the TCI and sunshine hours is 30% more than the TCI, which is due to the importance of these two variables in the location of development (Ma et al. 2020).

**Table 3:** All four climate indices with corresponding weigthings according to the climate variables included in their index equations

Index	Daytime thermal comfort	24-hour thermal comfort	Wind	Rain	Sunshine hours	Cloud Cover
TCI (Mieczkowski 1985)	40%	10%	10%	20%	20%	n/a
HCI_urban_ (Scott et al. 2016)	40%	n/a	10%	30%	n/a	20%
HCI_beach_ (Rutty et al. 2020)	20%	n/a	10%	20%	n/a	40%
CCI (Ma et al. 2020)	50%	*	*	*	50%	
* Included through threshold values for automatic scores of ‘unfavourable’ Min score (CCI; 3) if T_min_ <8 °C; or T_max_>34 °C; or *P* > 10 mm; or W > 23 km = h						
$\:\text{T}\text{C}\text{I}=4\text{c}\text{i}\text{d}+\text{c}\text{i}\text{a}+2\text{R}+2\text{S}+\text{W}$ HCI urban = 4(TC) + 2(A) + (3(precipitation) + wind) HCI Beach = 2(TC) + 4(A) +(3(P) + W) CCI = 0:5*TC + 0:5*S						

The key difference in the weightings is derived from where these indices had been developed and the specific locations and touristic environments for which they will be applied. In the South African context, the application of these indices is acceptable, considering the fact that individuals consider each variable from either one of the indices to be of relative importance (Fig. 5). Furthermore, at a national scale, the relative levels of importance ascribed to each of the meteorological values by tourists’ respondents in South Africa is consistent with the range of weightings in these indices, reflecting the sector-specific differences in weighting. However, when looking at a city-scale, or comparing local versus international tourists, the relative importance of the variables do differ. However, to produce different tourism climate index weightings per city would prevent comparison between cities within South Africa, or South Africa and the rest of the world. It is important to note that tourists may have pre-existing perceptions of weather conditions which may then affect their sensitivity to climate variables but also affect how they interpret certain experiences (Fitchett and Hoogendoorn 2018). Therefore, many of the findings of this indicated that certain climate variables did not matter to tourists unless they were directly affected by them. Finally, it is important to interrogate whether the intrinsic assumptions within each index hold true for the local context (Fitchett and Meyer 2023). For example, the omission of nighttime thermal comfort in the HCI_urban_ and HCI_beach_ is with the assumption that air-conditioning is universal, which is not the case globally or in South Africa, and thus this index should be used in instances where air conditioning has been confirmed to be present and operational (Mnguni and Fitchett 2025).

## The significance of meteorological thresholds

Temperature thresholds are an important determinant in the tourism location as tourists are least likely to travel to a destination that has a temperature mean above or below their threshold. This is also true for other climate variables like precipitation, windspeed and the number of sunshine and cloud cover hours. The thresholds also indicate whether a place is suitable for tourism or whether the certain activity can take place there.

In this study, the temperature thresholds recorded for South African tourists ranged from 10 °C for unacceptably cold and 30 °C to 35 °C for unacceptably hot (Fig. 3.4). When comparing these temperatures to the rankings given in the TCI classification table, conditions would range from being unfavourable to extremely unfavourable with a TCI score of 30–39 and 10–19 (Mieczkowski 1985). Following this, temperatures for daytime and night-time thermal comfort that exceed a threshold of 36 °C and range between − 6 °C and − 10 °C will receive an automatic zero as a TCI score, as weather conditions would be classified as impossible (Mieczkowski 1985).

Similarly, for the international tourists, there had been temperatures recorded which ranged from 10 °C for unacceptably cold to 30 °C for unacceptably hot (Fig. 3.5). Again, the rankings given in the TCI classification table compared to these temperatures will consider the weather conditions to be extremely unfavourable and unfavourable (Mieczkowski 1985).

When comparing these results to the scorings of other climate indices, like the HCI_urban_ and HCI_beach,_ the temperature thresholds do align with what the classification table deems as unacceptable or acceptable for tourism to take place (Scott et al. 2016; Rutty et al. 2020). For the HCI_urban_ and HCI_beach,_ an ideal temperature range would be between 20 and 26 °C, while unacceptable temperatures are between ≤ 15 and ≥ 31 °C (Scott et al. 2016).

Regarding the thresholds for precipitation, both local and international tourists agreed that between 0 and 20 h of rainfall is an unacceptable amount of consecutive rainfall (Fig. 6). When comparing these results to the TCI classification table, precipitation is measured in millimetres (mm), while our results show hours. This is because people are not able to perceive how much it rained in ‘mm’, but in hours. The TCI classification table does however indicate that an variable classification score of 9 is considered ideal weather whereas an classification score of 1 is extremely unfavourable, and 0 is considered impossible for tourism (Mieczkowski 1985). Therefore, precipitation between 0.00 and 0.49 mm is deemed ideal, where 4.50–4.99 mm is extremely unfavourable and ≥ 5.00 mm is impossible weather (Rutty et al. 2020).

For the precipitation, 0 mm is ideal, while ≥ 75 mm is unacceptable (Scott et al. 2016). With camping being both a tourist activity and a type of accommodation, certain climate variables may affect this drastically (Brooker and Joppe 2013). Therefore, if the climate variable surpasses any of the required limits for camping to take place, the CCI scoring will automatically be at a maximum score of 3 which means that the climate is unsuitable for camping (Ma et al. 2020; Table 3).

### Towards future evaluation of index suitability

The importance of determining the local suitability of tourism climate indices and tourists’ thresholds for suitable weather for tourism extends beyond factors such as physiological capability and sensitivity to the climate (Luther et al. 2016). Understanding tourists’ thresholds can help with seasonality-related tourism policy and planning. Seasonality is not solely based on the natural timing climate and the suitability of different climatic variables for tourism, but it is also based on the timing of social or religious events within a specific tourist destination (Amelung et al. 2007). In recent years, these institutional factors that lead to seasonality has raised many concerns in the space of policymaking, however it is still important to note that natural seasonality plays a crucial role in tourism (Amelung et al. 2007; Becken et al. 2020). The findings of this study demonstrate that these tourism climate indices are suitable for use in South Africa.

In addressing tourism operations related to policy making, climate variability that results in increasing or decreasing temperatures, affects thresholds, tourist comfort and safety, as well as tourism attractiveness, also needs to be considered (Scott and Lemieux 2010). We advocate for wider sectoral use of tourism climate indices, coupled with careful engagement with the thresholds for tourist comfort. Regarding this study, the climate variability in South Africa is reason for the multiple thresholds for one city as well as the differences in temperature thresholds, which promotes the encouragement of policy and tourism planning regarding the seasonality of tourist visitation to the country (Table 2). This therefore means that if temperatures go above a certain threshold, tourists may evidently receive a warning to stay indoors to avoid the risk of heat stress, and marketing and policy can influence avoiding travelling to a specific destination at a certain time of year, thereby affecting the seasonality of travelling (Gössling and Hall 2006; Curtis et al. 2011).

Furthermore, using questionnaire-based data, rather than Tripadvisor reviews allows tourists to directly consider the how they experience specific climate variables, rather than being directed by leading questions about their experience at a destination (Fitchett and Hoogendoorn 2018). However, Tripadvisor reviews give a more objective view of people’s experiences, without necessarily highlighting the importance of climate, and they are more accessible than handing out questionnaires in different cities. The use of web-scraping methods also allows for an easier way to analyse TripAdvisor reviews and obtain more accurate information (Mokgehle and Fitchett 2024).

## Conclusion

There is no one tourism climate index that can classify the climatic suitability of a given location as a range of touristic activities characterise a vacation, each requiring unique climatic conditions. This study analysed the use of questionnaire-based data to validate tourism climate indices in South Africa. Validating the TCI, in particular, using questionnaires was a necessity, because it had not been done before, but it also highlights the use of questions to guide people into thinking about weather conditions, they had experienced beforehand. The results of this study conclude that there is comparability between the TCI, HCI urban and beach and the CCI and that these indices can be used across countries and cities. Additionally, validation using both questionnaire-based data and Tripadvisor reviews is preferable as Tripadvisor reviews can be used for remote validation across countries and gives objective data however, questionnaires are more subjective to climate variables and climatic experiences. We find these indices are valid for application in South Africa without modification, provided that the intrinsic assumptions hold true for the specific activity, accommodation establishment and destination.

## Data Availability

In line with the ethics approval granted, these data are treated as confidential.
